# Technology for Quantifying the Postpartum Blood Loss

**DOI:** 10.17691/stm2020.12.3.09

**Published:** 2020-06-28

**Authors:** A.M. Ziganshin, V.A. Mudrov

**Affiliations:** Associate Professor, Department of Obstetrics and Gynecology; Bashkir State Medical University, 3 Lenin St., Ufa, Republic of Bashkortostan, 450008, Russia; Associate Professor, Department of Obstetrics and Gynecology, Faculties of Medicine and Dentistry; Chita State Medical Academy, 39a Gorky St., Chita, 672000, Russia

**Keywords:** uterine cavity, hypotonic postpartum hemorrhage, ultrasound of the postpartum uterus, 3D modeling, postpartum blood loss volume.

## Abstract

**The aim of the study** was to develop a technology for determining postpartum hemorrhage volume based on gravimetry and 3D modeling of the results of the US postpartum uterus examination.

**Materials and Methods.:**

The study was conducted at the perinatal center of the Regional Clinical Hospital (Chita) and the Kuvatov Republican Clinical Hospital (Ufa). A prospective analysis of 40 births from 2018–2019 was performed. Two groups were formed: group 1 — 30 women with physiological blood loss in the postpartum period; group 2 — 10 women with early hypotonic bleeding.

The volume of blood loss was determined in three ways: visually, by a gravimetric method, and by the integrated use of the gravimetric method and 3D modeling of the results of US of postpartum uterine cavity. Uterus sonography in the early postpartum period was performed with a MySono U6 portable ultrasound scanner (Samsung Medison, Korea). Using local systems for changing the positions of points, lines, and polygons, the Autodesk 3ds Max program was tuned to simulate the shape of the postpartum uterus, which changed dynamically in accordance to the input ultrasound parameters.

**Results.:**

In group 1, the volume of postpartum blood loss was quantified by the visual method as 275.0 (267.2; 282.8) ml, by the gravimetric method — as 375.0 (364.5; 388.2) ml, and by a combination of the gravimetric method and sonography-based 3D modeling of the postpartum uterus — as 420.0 (412.5; 435.4) ml. In group 2, the volume of postpartum blood loss was estimated visually as 725.0 (716.8; 773.2) ml, by gravimetry — as 1010.0 (1006.2; 1085.7) ml, and by gravimetry combined with the 3D modeling of the uterine cavity — as 1240.0 (1195.4; 1286.6) ml.

**Conclusion.:**

The proposed technology allows one to determine the volume of postpartum hemorrhage with a minimum error making it possible to reduce the frequency of massive postpartum bleeding and optimize the management of patients with medium and high risks.

## Introduction

Obstetric hemorrhages are among the top five causes of maternal mortality. More than 50,000 women die annually worldwide due to excessive blood loss; that is about 20% of the total maternal mortality. In the Russian Federation, obstetric bleeding takes the first place among the causes of death in the postpartum period [1, 2]. The severity of the clinical picture depends on the pace and duration of bleeding, as well as on the clinical condition of the woman. Massive bleeding is accompanied by the development of hemorrhagic shock and consumption coagulopathy with its aggravated clinical picture. However, by large, maternal deaths do not result from rapid and massive blood losses, but they are due to the ineffective treatment of wave-like prolonged bleeding of low intensity, in which the doctor logically delays taking strategically important decisions [1]. Therefore, in the management of these patients, special attention should be paid to a correct assessment of the amount of blood lost. The assessment of the volume and severity of blood loss is currently carried out either visually, or by gravimetry, or using clinical symptoms of the developing hypovolemia [2]. The visual method is inaccurate and gives about 30% error due to its subjectivity. This error increases with an increasing volume of bleeding [2–4]. The gravimetric method involves the direct collection of lost blood into graduated containers or the weighing of blood-soaked gauze pads. However, the accuracy of quantifying the volume of blood using the gravimetric method does not exceed 90% [2]. The management protocol of physiological birth does not take into account the blood clots retained in the uterine cavity in the early postpartum period; that often leads to an erroneous assessment of a real blood loss [5].

The absence of precise measurement tools necessitates a search into new up-to-date methods for the blood loss assessment. Among those, a dynamic measurement of the uterine cavity volume along with the gravimetric determination of blood lost (using a bag-collector) may be promising. To measure the uterus cavity volume, we chose the ultrasound method due to its exceptional diagnostic value, mobility, and safety of modern sonography devices [6].

**The aim of the study** was to develop a technology for determining postpartum hemorrhage volume based on gravimetry and 3D modeling of the results of the US postpartum uterus examination.

## Materials and Methods

A prospective analysis of 40 births delivered in the perinatal center of the Regional Clinical Hospital (Chita) and the Kuvatov Republican Clinical Hospital (Ufa) in 2018–2019 was carried out. Two study groups were formed: group 1 included 30 patients with physiological blood loss in the postpartum period; group 2 included 10 patients with early hypotonic bleeding. Patients with birth canal traumas accompanied by heavy bleeding as well as those with retention of placental tissue or primary bleeding disorders were excluded from the study.

To identify factors contributing to hypotonic bleeding, we analyzed the patients’ anamneses including gynecological and extragenital conditions, menstrual and reproductive functions. The groups were comparable by age, somatic pathology, and birth history. All women underwent a general and special obstetric examination in accordance with clinical recommendations approved by the Ministry of Health of the Russian Federation [2].

The volume of blood loss was quantified by three ways: visual, gravimetric, and a combination of gravimetry with 3D modeling of the postpartum uterine cavity based on the US examination results. With the development of postpartum hemorrhage, we used dynamic weighting of blood collected in a cylindrical container and weighting of blood-soaked pads as a reference method (the gold standard) for assessing the volume of blood loss.

The work was carried out in accordance with the requirements of the Helsinki Declaration of the World Health Organization (2013) and approved by the Ethics Committee of the Bashkir State Medical University. Prior to the study, informed consent was obtained from all patients.

*The first stage* of the study was the development of the formula to determine the volume of the postpartum uterus cavity.

In ultrasound practice, the volume of a hollow organ (*V*, cm^3^) is determined by the formula: *V=ABC***·**0.523, where *A* is the length (cm); *B* — thickness (cm); *C* is the width (cm) [7, 8].

Since the uterus cavity has an irregular geometric shape (in particular, when the uterus is deformed by a myomatous node), its volume is estimated from the cross-sectional area of the cavity [9, 10].

The cross-sectional area of an ellipsoid is *S=abπ*, where *a* is the first (major) half-axis; *b* — second (middle) half-axis. The area of an ellipse is defined as follows: *S=AB*π/4, where *A* is the first (major) axis (organ length); *B* — second (middle) axis (organ thickness).

Then the length of this ellipsoid image is *A=*4*S*_1_/*B*π, where *S*_1_ is the area of the longitudinal section; the width of this ellipsoid is *С=*4*S*_2_/*B*π, where *S*_2_ is its cross-sectional area.

Thus, the volume of the ellipsoidal cavity can be calculated from the formula:

*V =* (4*S*_1_/*B*π) (4*S*_2_ /*B*π)*B***·**0.523 = 0.8487*S*_1_*S*_2_ /*B*.

Therefore, the volume of the postpartum uterus cavity can be determined by the formula: *V=*0.8487*S*_1_*S*_2_/*B*, where *S*_1_ is the area of the maximum longitudinal section of the cavity (cm^2^); *S*_2_ — the cross-sectional area of the cavity (cm^2^); *B* — the maximum anteroposterior size of the cavity (cm).

**Figure 1:**
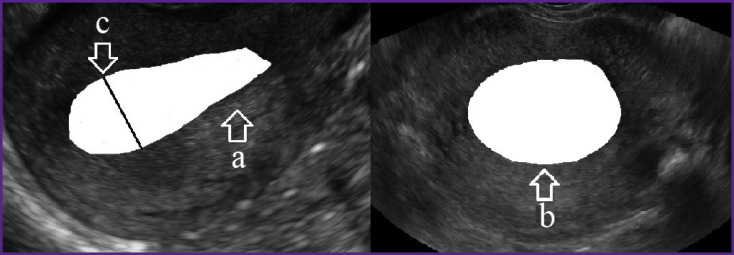
The maximum longitudinal (a), cross (b) sections, and anteroposterior size (c) of the postpartum uterus cavity as determined by ultrasound image tracing

Sonography of the uterus in the early postpartum period was performed using a high-class portable ultrasound scanner MySono U6 (Samsung Medison, Korea) with a transabdominal probe frequency of 3.5–5.0 MHz. The measurement was carried out when the patient was in supine position. The convex transducer was placed in the sagittal position on the paraumbilical segment to visualize the uterine fundus. Then, using the “search mode”, we obtained an image of the longitudinal section of the uterine cavity of the maximum possible size. Switching to the “freeze frame”, we determined the area of the maximum longitudinal section of the cavity; then, using the tracing mode we measured the maximum anteroposterior size of the cavity (Figure 1 (a), (c)). After that, at the same visualization point, the probe was rotated 90° to obtain a cross-section of the uterus cavity and determine its size by the tracing method (Figure 1 (b)).

**Figure 2:**
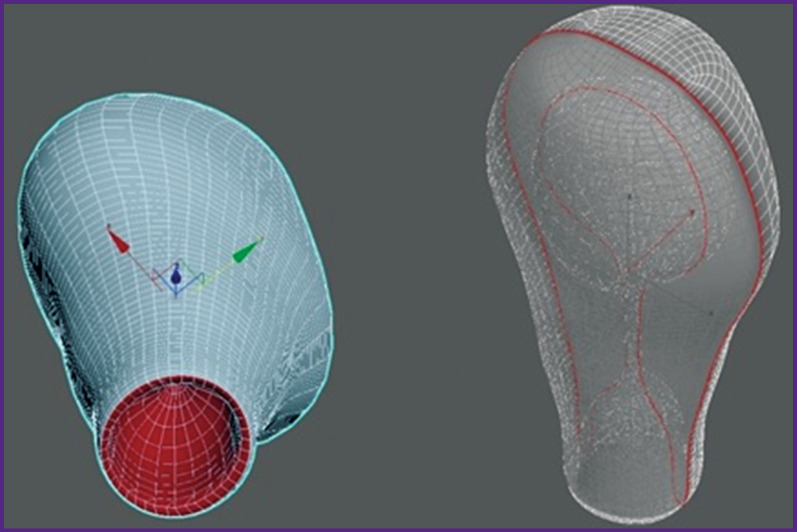
Creating a 3D model of the postpartum uterus cavity

**Figure 3:**
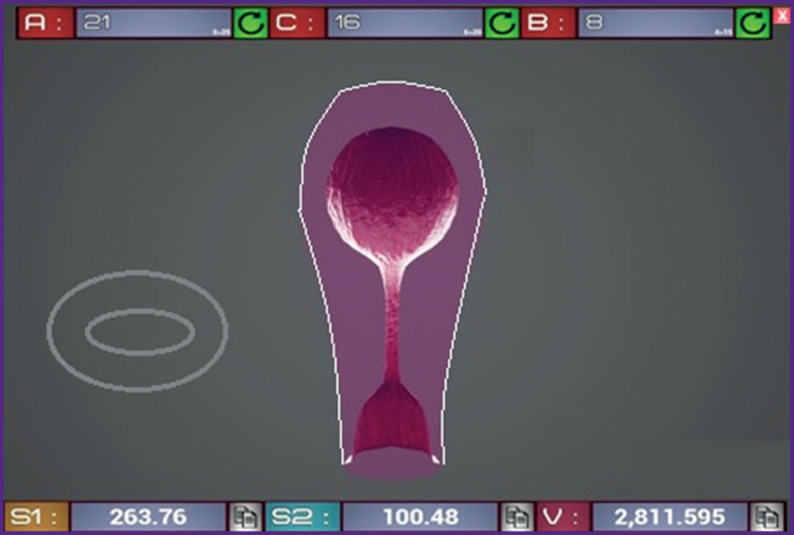
The working windows of the developed program and the 3D model of the postpartum uterus (top view)

*The second stage* of the study was to create a 3D model of the postpartum uterus cavity using the ultrasound data [10]. With the help of the local systems for changing the positions of points, lines, and polygons, the Autodesk 3ds Max [11] was tuned to simulate the shape of the postpartum uterus (Figure 2). The model resembles a virtual shell that changes its shape according to the input ultrasound data. Based on the obtained model, a program code was scripted using the Unreal Engine 4 Blueprint visual scripting system based on C++. When the program starts, a dialog box for entering ultrasound data opens. The following measures have to be entered: length (*A*), width (*C*), maximum anteroposterior size (*B*), maximum longitudinal area (*S*_1_), and cross-section area (*S*_2_) of the postpartum uterus cavity (Figure 3).

The subsequent actions are triggered automatically in the user window. The result is displayed as a volume of the uterus cavity (*V*) and a color scale indicating the severity of the blood loss: 100–1000 ml — green, 1000–1500 ml — yellow, 1500–2000 ml — blue, and 2000–3000 ml — red.

The input window and the projection window interact with each other by relating the variables of the input window to the polygonal sections of the model [12].

Our original software package appended to this 3D model allowed us to calculate the true volume of postpartum hemorrhage and also estimate the volume of external blood loss by the gravimetric method. This way, one can optimize patient management and prevent the development of critical conditions.

### Statistical data processing.

Statistical processing of the results was carried out using the software package IBM SPSS Statistics v. 25.0. Taking into account the number of studied groups (less than 50), the data distribution normality was assessed using the Shapiro–Wilk test. The results indicated that the data in both groups did not obey the law of normal distribution. Therefore, we decided to present the results as median values, the first and third quartiles: Me [Q_1_; Q_3_]. To compare the groups by one quantitative criterion, the Mann–Whitney test (U) was used. The Kruskal–Wallis (H) rank analysis of variance was used to compare the methods for estimating postpartum blood loss. Then, if significantly different, a pairwise comparison was performed using the Mann–Whitney test with the Bonferroni correction. In all cases, p<0.05 was considered statistically significant [13].

## Results and Discussion

In group 1, the mean weight of fetuses at birth was 3285.0 (3186.8; 3346.5) g, and in group 2 — 3530 (3364.2; 3641.9) g (U=97.5; p=0.1). The absence of significant differences between the groups is probably due to the small number of patients. Comparing the frequency of complications in the intrapartum period did not make sense for the same reason.

In group 1, the volume of postpartum blood loss estimated by the visual method was 275.0 (267.2; 282.8) ml, by gravimetry — 375.0 (364.5; 388.2) ml, and by the gravimetric method combined with the 3D modeling of the postpartum uterine cavity based on the US examination results — 420.0 (412.5; 435.4) ml (H=57.55; df=2; p<0.001). Obviously, the third approach is the most objective in this case, since it allows us to evaluate not only the volume of external blood loss, but also the volume of blood remained in the uterine cavity. The visual method underestimates the volume of total blood loss in group 1 by 145.0 (136.3; 152.6) ml (U=6.0; p<0.001) and the gravimetric method — by 45.0 (44.0; 47.2) ml (U=6.0; p<0.001). It was not possible to directly measure the amount of uterus-deposited blood since there were no indications for manual examination of the uterine cavity.

**Figure 4:**
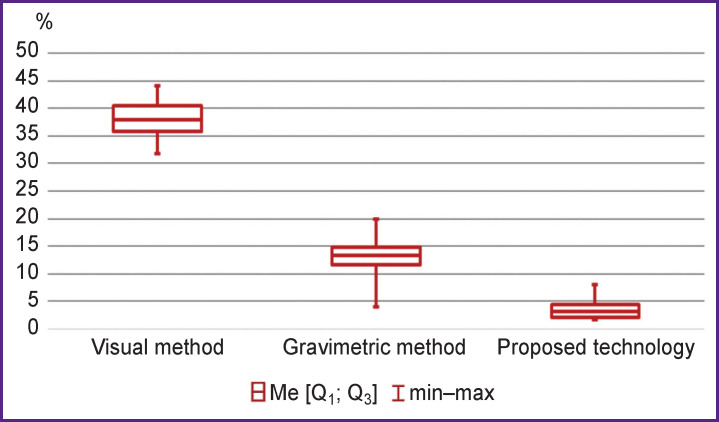
The relative errors of the compared methods for quantifying the blood loss volume

In group 2, the volume of postpartum blood loss equal to 725.0 (716.8; 773.2) ml was estimated by the visual method, 1010.0 (1006.2; 1085.7) ml by the gravimetric method, and 1240.0 (1195.4; 1286.6) ml by the gravimetric method combined with the 3D modeling of the postpartum uterine cavity based on the US examination results; the reference method produced the result of 1200.0 (1159.6; 1242.4) ml (H=28.64; df=3; p<0.001). With the development of postpartum hemorrhage, the visual method underestimates the amount of blood loss by 465.0 (430.6; 481.4) ml (U=0.0; p<0.001) and the gravimetric method — by 150.0 (133.5; 176.5) ml (U=14.0; p=0.005). Along with that, the proposed technology overestimates the true volume of blood loss by 40.0 (27.7; 52.3) ml, which is not statistically significant (U=39.0; p=0.44). Therefore, the combined use of the gravimetric method and 3D modeling of a postpartum uterus based on the US examination results allows us to determine the true volume of postpartum blood loss with a minimum error. The overestimation of the blood loss is probably due to friability of blood clots deposited in the cavity of the postpartum uterus. The assessment of the absolute error of the compared methods did not allow us to clearly demonstrate any significant differences between the two groups. Therefore, we decided to determine the relative error of the methods defined as the ratio of the absolute error of the given method to the volume of true blood loss obtained by the reference method (Figure 4).

Thus, the relative error of the visual method was higher than the error of the gravimetric method by almost 3 times (U=0.0; p<0.001) and than the error of the developed technology by 10 times (U=0.0; p<0.001). Notably, the technique of 3D modeling of the postpartum uterus cavity allows for a statistically significant decrease in the relative error of the gravimetric method by 3.5 times (U=4.0; p<0.001).

The present results demonstrate a high accuracy of the proposed technology for assessing the blood loss volume. The method is recommended for use in the cases of suspected early postpartum hemorrhage (women of medium and high risk) [2].

## Conclusion

The combination of 3D modeling of the sonography data on the uterus cavity in the early postpartum period and the gravimetric method for assessing the volume of external blood loss makes it possible to determine the volume of postpartum blood loss with a minimum error. The proposed technology makes it possible to reduce the frequency of massive postpartum bleeding and optimize the management of patients.

## References

[1] Radzinskiy V.E., Fuks A.M. (2016). Akusherstvo.

[2] (2019). Profilaktika, algoritm vedeniya, anesteziya i intensivnaya terapiya pri poslerodovykh krovotecheniyakh. Klinicheskie rekomendatsii.

[3] Mavrides E., Allard S., Chandraharan E., Collins P., Green L., Hunt B., Riris S., Thompson A. (2017). Prevention and management of postpartum haemorrhage.. BJOG An Int J Obstet Gynaecol.

[4] Lilley G., Burkett-St-Laurent D., Precious E., Brunseels D., Kaye A., Sanders J., Alikhan R., Collins P.W., Hall J.E., Collis R.E. (2015). Measurement of blood loss during postpartum haemorrhage.. Int J Obstet Anesth.

[5] (2014). Okazanie meditsinskoy pomoshchi pri odnoplodnykh rodakh v zatylochnom predlezhanii (bez oslozhneniy) i v poslerodovom periode. Klinicheskie rekomendatsii.

[6] Eberhard M. (2016). Ul’trazvukovaya diagnostika v akusherstve i ginekologii. T. 1. Akusherstvo.

[7] Smith N.C., Smith E.P.M. (2014). Ul’trazvukovaya diagnostika v akusherstve i ginekologii ponyatnym yazykom.

[8] Zmitrovich O.A. (2014). Ul’trazvukovaya diagnostika v tsifrakh.

[9] Kolesov V.V. (2015). Matematika dlya meditsinskikh vuzov.

[10] Remizov A.N. (2012). Meditsinskaya i biologicheskaya fizika.

[11] Autodesk team (2007). Ofitsial’nyy kurs obucheniya paketu 3ds max.

[12] Ziganshin A.M., Mudrov V.A., Lyapunov A.K. (2019). Three-dimensional simulated system for estimating the volume of early hypotonic bleeding..

[13] Lang T.A., Altman D.G. (2014). Basic statistical reporting for articles published in biomedical journals: the “statistical analyses and methods in the published literature” or the SAMPL guidelines.. Int J Nurs Stud.

